# Pembrolizumab plus epacadostat in patients with recurrent/metastatic head and neck squamous cell carcinoma (KEYNOTE-669/ECHO-304): a phase 3, randomized, open-label study

**DOI:** 10.1186/s12885-023-11316-0

**Published:** 2024-07-25

**Authors:** Byoung Chul Cho, Irene Braña, Beatriz Cirauqui, Sercan Aksoy, Felix Couture, Ruey-Long Hong, Wilson H. Miller, Manuel Chaves-Conde, Margarida Teixeira, Lance Leopold, Mihaela Munteanu, Joy Yang Ge, Ramona F. Swaby, Brett G. M. Hughes

**Affiliations:** 1 Division of Medical Oncology, Yonsei Cancer Center, Yonsei University College of Medicine, 50-1 Yonsei-Ro, Seoul, 03722 Republic of Korea; 2 Severance Hospital and Yonsei University, 50-1 Yonsei-Ro, Seoul, Korea; 3 Medical Oncology Department, Vall d’Hebron University Hospital, Vall d’Hebron Institute of Oncology (VHIO), Barcelona, 08035 Spain; 4 Oncology Department, Catalan Institut of Oncology (ICO) Badalona, Crta Canyet sn, Barcelona, 08916 Spain; 5 Hacettepe University Cancer Institute, Hacettepe Mh., Ankara, 06100 Turkey; 6 CHU de Québec – Hôtel-Dieu de Québec, 11 Cote du Palais, Quebec City, QC G1R 2J6 Canada; 7 National Taiwan University Hospital, No.1, Changde St., Zhongzheng District, Taipei, 100 Taiwan; 8 Jewish General Hospital and McGill University, 3755 Cote St., Montreal, H3S 1Z1 Canada; 9 Hospital de Nuestra Senora de Valme, Ctra Cadiz sn, Seville, 41014 Spain; 10 Instituto Português de Oncologia de Coimbra Francisco Gentil EPE, Avenida Bissaya Barreto 98, Coimbra, 3000-075 Portugal; 11 Incyte Corporation, Wilmington, DE 19803 USA; 12 Merck & Co., Inc., P.O. Box 2000, 126 East Lincoln Ave., Rahway, NJ 07065 USA; 13 Royal Brisbane and Women’s Hospital and University of Queensland, Butterfield Street, Ground Floor, Building 34, Brisbane, QLD 4029 Australia

**Keywords:** Pembrolizumab, PD-1, Immunotherapy, EXTREME, Cetuximab, Head and neck squamous cell carcinoma

## Abstract

**Background:**

Advanced head and neck squamous cell carcinoma (HNSCC) has a poor prognosis, and new treatment options are needed. Combining immunotherapies with differing mechanisms of action may enhance clinical benefits compared with single-agent immunotherapy. Epacadostat, an indoleamine 2,3 dioxygenase 1 inhibitor, plus pembrolizumab, a PD-1 inhibitor, showed promising activity in advanced HNSCC in the phase 1/2 KEYNOTE-037/ECHO-202 trial.

**Methods:**

KEYNOTE-669/ECHO-304 is a randomized, open-label, phase 3 study evaluating the efficacy and safety of pembrolizumab plus epacadostat, pembrolizumab monotherapy, and the EXTREME regimen (cetuximab with a platinum [carboplatin or cisplatin] and 5-fluorouracil) in recurrent/metastatic (R/M) HNSCC. Participants had no prior systemic therapy for R/M HNSCC and were randomly assigned (2:1:2) to pembrolizumab 200 mg intravenously every 3 weeks plus epacadostat 100 mg orally twice daily, pembrolizumab monotherapy, or EXTREME. The primary endpoint was objective response rate (ORR; investigator assessment). Secondary endpoints were safety and tolerability. Change in serum kynurenine was an exploratory endpoint. Study enrollment was discontinued early as a strategic decision on May 2, 2018, and response assessment was discontinued after first on-study imaging assessment at week 9. Data cut-off was January 17, 2019.

**Results:**

Between December 1, 2017, and May 2, 2018, 89 patients were randomly allocated to pembrolizumab plus epacadostat (*n* = 35), pembrolizumab monotherapy (*n* = 19), or EXTREME (*n* = 35). ORR (95% CI) was 31% (17%–49%) for pembrolizumab plus epacadostat, 21% (6%–46%) for pembrolizumab monotherapy, and 34% (19%–52%) for EXTREME. Treatment-related adverse events (TRAEs) occurred in 82% (*n* = 28) of patients receiving pembrolizumab plus epacadostat, 63% (*n* = 12) receiving pembrolizumab monotherapy, and 100% (*n* = 34) receiving EXTREME. Grade 3–4 TRAEs occurred in 24% (*n* = 8) of patients receiving pembrolizumab plus epacadostat, 16% (*n* = 3) receiving pembrolizumab monotherapy, and 82% (*n* = 28) receiving EXTREME. No deaths occurred due to AEs. Pembrolizumab plus epacadostat treatment reduced kynurenine levels but not to that of healthy subjects.

**Conclusions:**

Pembrolizumab plus epacadostat and pembrolizumab monotherapy provided a similar response rate to EXTREME and demonstrated a manageable safety profile in patients with R/M HNSCC.

**Trial registration:**

NCT03358472. Date of trial registration: November 30, 2017.

**Supplementary Information:**

The online version contains supplementary material available at 10.1186/s12885-023-11316-0.

## Background

Head and neck squamous cell carcinomas (HNSCC) arise from the epithelial cells that line the oral cavity, pharynx, and larynx [1, 2]. More than 890,000 cases of HNSCC are diagnosed each year and result in 450,000 annual deaths worldwide [1]. The majority of patients are diagnosed with locoregionally advanced disease, for whom recurrence and distant metastasis are common [1]. The prognosis for patients with recurrent or metastatic (R/M) disease is poor, with a median overall survival (OS) of only 10–13 months [3]. HNSCC can also have devastating effects on patient quality of life, including impaired basic functions, social isolation, and potential disfigurement, all of which point to the value of preventing progression in this patient population [4].

The programmed death 1 (PD-1) inhibitor pembrolizumab has demonstrated acceptable safety and effective antitumor activity in patients with R/M HNSCC [5–7]. In the KEYNOTE-040 study, pembrolizumab monotherapy in previously treated patients demonstrated a clinically meaningful prolongation of OS, a similar response rate, and a manageable safety profile compared with investigator’s choice of standard of care (cetuximab, docetaxel, or methotrexate) [5]. In the KEYNOTE-048 study enrolling treatment-naive patients with incurable R/M disease, pembrolizumab monotherapy significantly improved OS compared with EXTREME (cetuximab in combination with platinum-based chemotherapy and 5-fluorouracil [5-FU]) in the programmed death ligand-1 (PD-L1) combined positive score (CPS) ≥ 20 and CPS ≥ 1 populations with noninferior OS in the total population, and provided lower response rates and manageable safety [7]. Pembrolizumab in combination with chemotherapy significantly improved OS in the CPS ≥ 20, and CPS ≥ 1, and total populations and demonstrated similar response rates and comparable safety compared with the EXTREME treatment regimen [7].

Current first-line standard of care for patients with recurrent, unresectable, or metastatic HNSCC (non-nasopharyngeal) not amenable to surgery or radiation therapy includes pembrolizumab monotherapy in patients with tumors expressing PD-L1 CPS ≥ 1, pembrolizumab in combination with platinum-based chemotherapy and 5-FU, or the EXTREME regimen [8]. However, use of EXTREME or EXTREME-based regimens may need to be restricted to specific patients because of the tolerability profile and limited therapeutic benefit of cetuximab demonstrated in elderly patients or those with poor performance status [9].

Epacadostat is a novel, potent, and highly selective oral inhibitor of the indoleamine 2,3 dioxygenase 1 (IDO1) enzyme [10]. IDO1 catabolism of tryptophan to kynurenine inhibits T-cell-mediated immune responses and IDO1 expression has been shown to be elevated in many cancers [11]. An IDO1 inhibitor may restore an effective antitumor immune response. In a phase 1 study in patients with advanced solid malignancies, epacadostat monotherapy showed normalization of kynurenine levels and was generally well tolerated [12]. Combining immune checkpoint inhibitors with therapies that have different mechanisms of action has the potential to further enhance the clinical benefits of single-agent immunotherapy [13]. Preliminary data for pembrolizumab in combination with epacadostat in patients with advanced solid tumors who received 1 or more prior lines of therapy in the phase 1/2 KEYNOTE-037/ECHO-202 trial showed objective responses in 40% of patients with solid tumors, including in one of two patients with HNSCC [14]. Treatment with the combination was generally well tolerated; grade ≥ 3 treatment-related AEs occurred in 11% of patients with advanced solid tumors (*n* = 62). In this paper, we report the efficacy and safety of pembrolizumab in combination with epacadostat compared with pembrolizumab monotherapy or EXTREME in patients with R/M HNSCC. The current trial was modified as a strategic decision following the results of an interim analysis of the KEYNOTE-252/ECHO-301 trial of pembrolizumab and epacadostat versus pembrolizumab alone in patients with advanced melanoma [15]. The data monitoring committee of KEYNOTE-252 found that although no safety issues were identified, the combination of pembrolizumab and epacadostat was unlikely to improve the progression-free survival (PFS) or OS (primary endpoints) versus pembrolizumab alone in patients with advanced melanoma [15]. We present the final results of the KEYNOTE-669/ECHO-304 trial.

## Methods

### Study design and patients

KEYNOTE-669/ECHO-304 was a randomized, active-controlled, multi-site, open-label, phase 3 study evaluating 3 parallel groups: pembrolizumab plus epacadostat, pembrolizumab monotherapy, and the EXTREME regimen. This study was conducted at 76 centers in 14 sites globally (Supplementary Table 1). Eligible patients were aged ≥ 18 years with histologically or cytologically-confirmed R/M HNSCC that was considered incurable by local therapies. Patients with primary tumors of the oropharynx, oral cavity, hypopharynx, and larynx were eligible and were required to have measurable disease based on Response Evaluation Criteria in Solid Tumors version 1.1 (RECIST v1.1) by site radiology. Lesions situated in a previously irradiated area were considered measurable if progression had been demonstrated following radiation therapy. Additional eligibility criteria were Eastern Cooperative Oncology Group (ECOG) performance status 0 or 1; adequate organ function; known human papillomavirus (HPV) status for oropharyngeal cancer defined as p16 immunohistochemistry testing using the CINtec p16 histology assay and a 70% cut-off point; and newly obtained biopsy or archival tumor specimen. Exclusion criteria were carcinoma of the nasopharynx, salivary gland, unknown primary origin, or non-squamous histology as primary tumor; disease progression within 6 months of completion of curatively intended systemic treatment for locoregionally advanced HNSCC; life expectancy < 3 months; and receipt of prior systemic therapy for HNSCC in the R/M setting. The trial was conducted in accordance with Good Clinical Practice, and the protocol was approved at all sites by institutional review boards. All patients provided written informed consent to participate. This trial was registered at ClinicalTrials.gov (NCT03358472) on November 30, 2017.

### Treatment and assessments

Patients were randomly allocated in a 2:1:2 ratio into 3 treatment groups. The first group received pembrolizumab 200 mg intravenous (IV) infusion every 3 weeks (Q3W) for ≤ 35 cycles and epacadostat 100 mg orally twice daily for ≤ 35 cycles. The second group received pembrolizumab 200 mg IV infusion Q3W for ≤ 35 cycles. The third group received the EXTREME regimen, which included cetuximab 400 mg/m^2^ IV infusion given on cycle 1, day 1, followed by cetuximab 250 mg/m^2^ IV infusion every week until disease progression or unacceptable toxicity, in combination with platinum-based chemotherapy (cisplatin 100 mg/m^2^ IV infusion or carboplatin area under the curve 5 [AUC 5] IV infusion Q3W for ≤ 6 cycles) and 5-FU 1000 mg/m^2^/day continuous IV infusion over days 1–4 Q3W for ≤ 6 cycles. Randomization occurred centrally using an interactive voice response system/integrated web response system and was stratified according to ECOG performance status (0 vs 1), HPV p16 status (oropharynx-p16-positive vs oropharynx-p16-negative or larynx/hypopharynx/oral cavity HNSCC), and prior systemic oncological therapy for locally advanced disease (yes vs no). Patients received their allocated study treatment until disease progression, unacceptable adverse events (AE), investigator’s decision to withdraw patient, or receipt of 35 cycles of pembrolizumab plus epacadostat or pembrolizumab monotherapy. AEs were graded per the National Cancer Institute Common Terminology Criteria for Adverse Events, version 4.0, and were monitored throughout the study and for 30 days after treatment discontinuation (90 days for serious AEs). Blood samples for pharmacodynamic analysis were drawn before administration of any study drug on day 1 of cycle 1 and day 1 of cycle 2. Circulating kynurenine levels were determined using a proprietary, validated liquid chromatography–mass spectrometry assay using calibrated standards at Worldwide Clinical Trials, Morrisville, NC.

### Statistical analyses

Based on the findings of the data monitoring committee of KEYNOTE-252 (NCT02752074), the scope of this study was reduced to the collection of preliminary efficacy data (see Supplementary Methods for a summary of protocol amendments). PFS, OS, and duration of response were removed as endpoints and evaluation of objective response rate (ORR) became the primary objective. Efficacy endpoints were assessed only until week 9; safety endpoints remained unchanged. ORR was defined as the proportion of patients with a best response of complete response (CR) or partial response (PR). Responses were based on RECIST v1.1 as assessed by the investigator without confirmation using all available imaging assessments after the last patient completed the week 9 imaging assessment. Secondary endpoints were safety and tolerability. Change from baseline in circulating kynurenine was included as an exploratory endpoint. Circulating kynurenine levels were assessed using blood samples provided by patients on day 1 of cycle 1 and day 1 of cycle 2, and statistical comparisons were conducted using a paired Student’s *t* test within each treatment arm.

The sample size was approximately 90 patients with 36 each in the pembrolizumab plus epacadostat and EXTREME groups, and 18 in the pembrolizumab monotherapy group. ORR was estimated by treatment group; 95% confidence intervals (CIs) were assessed using the Clopper and Pearson exact method for binomial data. Safety and tolerability were assessed by clinical review of all relevant parameters, including AEs, laboratory tests, vital signs, and electrocardiogram measurements. The intention-to-treat population was used for the efficacy analysis and comprised all randomly allocated patients. The safety population comprised all randomly allocated patients who received ≥ 1 dose of study treatment. Results from the final efficacy analysis for ORR are presented. Database cut-off was January 17, 2019.

Study enrollment was discontinued early as a strategic decision (May 2, 2018) based on the findings of the external Data Monitoring Committee for KEYNOTE-252, which determined that pembrolizumab plus epacadostat versus pembrolizumab plus placebo in unresectable or metastatic melanoma did not significantly improve PFS and was not expected to significantly improve OS [15]. The KEYNOTE-669 protocol was subsequently amended (see Supplementary Methods for a summary of key protocol amendments), and response assessment for primary endpoint analysis was discontinued after the first on-study imaging at week 9 in all study treatment groups. Thereafter, patients were treated per standard of care for the disease and local guidelines. All efficacy procedures after week 9 (first on study scan for HNSCC study) were discontinued and disease monitoring was continued per standard of care. Patients were given the option to discontinue the study or continue study treatment. Safety procedures continued per protocol for all patients in the pembrolizumab-epacadostat group, the pembrolizumab monotherapy group; for patients in the EXTREME group, safety procedures continued per standard of care.

## Results

**Fig. 1:**
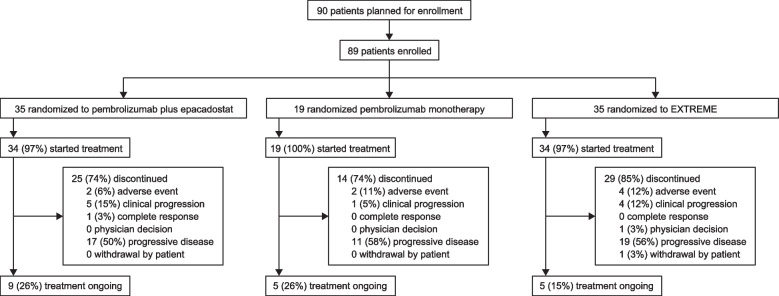
Patient disposition. ^a^1 patient was randomly assigned to the pembrolizumab plus epacadostat arm on May 2, 2018, but did not receive treatment because the strategic decision was made to stop patient enrollment on the same day. ^b^1 patient was randomly assigned to the EXTREME arm but did not receive EXTREME treatment because the patient withdrew consent to participate in the trial

Between December 1, 2017, and May 2, 2018, a total of 89 patients were enrolled and randomly allocated to receive pembrolizumab plus epacadostat (*n* = 35), pembrolizumab monotherapy (*n* = 19), or EXTREME (*n* = 35) (Fig. 1). Patient baseline characteristics were as expected and similar between treatment groups (Table 1). Median age was 64.0 years, and the majority of patients were male (84%), had an ECOG performance status of 1 (57%), had metastatic disease (81%), had previous radiation (78%), and had previously received systemic therapy (55%). Compared with the pembrolizumab plus epacadostat and the EXTREME arms, a greater proportion of patients in the pembrolizumab monotherapy group had an ECOG performance status of 1 (68% pembrolizumab monotherapy vs 54% pembrolizumab plus epacadostat vs 54% EXTREME), and more patients had recurrent versus metastatic disease (32% vs 17% vs 14%). Compared with the pembrolizumab plus epacadostat (11%) and the pembrolizumab monotherapy (16%) arms, a greater proportion of patients in the EXTREME arm (26%) had hypopharynx cancer. Compared with the pembrolizumab monotherapy arm, fewer patients in the pembrolizumab plus epacadostat group had oropharyngeal p16 positive disease (17% pembrolizumab plus epacadostat vs 26% pembrolizumab monotherapy) and fewer had received prior radiation therapy (66% vs 89%). More patients in the pembrolizumab plus epacadostat arm had PD-L1 CPS ≥ 1 (83%) and CPS ≥ 20 (43%) and more were current smokers (23%) compared with patients in the pembrolizumab monotherapy (CPS ≥ 1, 58%; CPS ≥ 20, 32%; current smokers, 11%) and EXTREME arms (CPS ≥ 1, 69%; CPS ≥ 20, 37%; current smokers, 9%).

**Table 1:** Baseline characteristics

**Characteristic**	**Pembrolizumab + Epacadostat** ***n* = 35**	**Pembrolizumab Monotherapy** ***n* = 19**	**EXTREME** ***n* = 35**
Median age, years (range)	64.0 (39–79)	62.0 (39–77)	63.0 (35–83)
Male, n (%)	30 (86)	16 (84)	29 (83)
Region of enrollment, n (%)			
North America	9 (26)	1 (5)	7 (20)
European Union	12 (34)	11 (58)	12 (34)
Rest of world	14 (40)	7 (37)	16 (46)
ECOG PS, n (%)			
0	16 (46)	6 (32)	16 (46)
1	19 (54)	13 (68)	19 (54)
Smoking status, n (%)			
Never	0	2 (11)	9 (26)
Former	27 (77)	15 (79)	23 (66)
Current	8 (23)	2 (11)	3 (9)
Oropharyngeal p16 positive, n (%)	6 (17)	5 (26)	8 (23)
PD-L1 status,^a^ n (%)			
CPS ≥ 1	29 (83)	11 (58)	24 (69)
CPS < 1	4 (11)	3 (16)	7 (20)
CPS ≥ 20	15 (43)	6 (32)	13 (37)
CPS < 20	18 (51)	8 (42)	18 (51)
Primary tumor site, n (%)			
Oropharynx	12 (34)	6 (32)	12 (34)
Oral cavity	10 (29)	5 (26)	7 (20)
Larynx	9 (26)	5 (26)	7 (20)
Hypopharynx	4 (11)	3 (16)	9 (26)
Cancer stage at baseline, n (%)			
IVA	3 (9)	3 (16)	5 (14)
IVB	4 (11)	2 (11)	0
IVC	28 (80)	14 (74)	30 (86)
Disease presentation at baseline, n (%)			
Metastatic	29 (83)	13 (68)	30 (86)
Recurrent	6 (17)	6 (32)	5 (14)
Prior radiation therapy, n (%)	23 (66)	17 (89)	29 (83)
Prior systemic therapy, n (%)	20 (57)	12 (63)	17 (49)

*CPS* Combined positive score, *ECOG PS* Eastern Cooperative Oncology Group performance status, *PD-L1* Programmed death-ligand 1

^a^PD-L1 status was not available for 11 patients (2 in pembrolizumab + epacadostat, 5 pembrolizumab monotherapy, and 4 in EXTREME)

Median time from randomization to data cut-off (January 17, 2019) was 9.5 months (range, 8.1–13.1). In the pembrolizumab plus epacadostat group, 34 patients were treated; 25 (74%) discontinued and 9 (26%) continued to receive study treatment. In the pembrolizumab monotherapy group, 19 patients were treated; 14 (74%) discontinued and 5 (26%) continued to receive study treatment. In the EXTREME group, 34 patients were treated; 29 (85%) discontinued and 5 (15%) continued to receive study treatment. The most common reasons for discontinuation of study treatment in all groups were progressive disease, clinical progression, and adverse events (Fig. 1).

ORR was 31% (11/35; 95% CI, 17%–49%) for the pembrolizumab plus epacadostat group, 21% (4/19; 95% CI, 6%–46%) for the pembrolizumab monotherapy group, and 34% (12/35; 95% CI, 19%–52%) for the EXTREME group (Table 2). CRs were achieved by 3 patients in the pembrolizumab plus epacadostat group and 2 patients in the EXTREME group. PRs were observed in 8 patients in the pembrolizumab plus epacadostat group, 4 patients in the pembrolizumab monotherapy group, and 10 patients in the EXTREME group. For patients with PD‑L1 CPS ≥ 1, ORR was 34% (10/29; 95% CI, 18%–54%) for the pembrolizumab plus epacadostat group (3 CR, 7 PR), 18% (2/11; 95% CI, 2%–52%) for the pembrolizumab monotherapy group (0 CR, 2 PR), and 29% (7/24; 95% CI, 13%–51%) for the EXTREME group (2 CR, 5 PR). For patients with PD‑L1 CPS ≥ 20, ORR was 40% (6/15; 95% CI, 16%–68%) for the pembrolizumab plus epacadostat group (1 CR, 5 PR), 33% (2/6; 95% CI, 4%–78%) for the pembrolizumab monotherapy group (0 CR, 2 PR), and 31% (4/13; 95% CI, 9%–61%) for the EXTREME group (2 CR, 2 PR). Due to the early closure of the study, no survival data are available.

**Table 2:** Summary of objective response based on investigator assessment per RECIST v1.1 in intention-to-treat population

	**Pembrolizumab + Epacadostat** ***n* = 35**	**Pembrolizumab Monotherapy** ***n* = 19**	**EXTREME** ***n* = 35**
Objective response rate, n (%) [95% CI]	11 (31) [17–49]	4 (21) [6–46]	12 (34) [19–52]
Best overall response, n (%)			
Complete response	3 (9)	0	2 (6)
Partial response	8 (23)	4 (21)	10 (29)
Stable disease	8 (23)	6 (32)	15 (43)
Progressive disease	13 (37)	9 (47)	5 (14)
No assessment^a^	3 (9)	0	3 (9)

Responses are based on investigator assessments per RECIST v1.1 without confirmation using all available scans

*CI* Confidence interval, *RECIST v1.1* Response Evaluation Criteria in Solid Tumors version 1.1

^a^No post-baseline assessments were available for response evaluation

The median pembrolizumab exposure was 87 days (range, 43–357) in the pembrolizumab monotherapy group and 127 days (range, 1–315) in the pembrolizumab plus epacadostat group. Median exposure to the EXTREME regimen was 130.5 days (range, 5–332). Median epacadostat exposure was 148.5 days (range, 10–335) in the pembrolizumab plus epacadostat group. AEs of any cause were experienced by 34 patients (100%) in the pembrolizumab plus epacadostat group, 17 (89%) in the pembrolizumab monotherapy group, and 34 (100%) in the EXTREME group (Tables 3 and 4). Grade 3–4 AEs of any cause occurred in 16 patients (47%) in the pembrolizumab plus epacadostat group, 13 (68%) in the pembrolizumab monotherapy group, and 29 (85%) in the EXTREME group.

**Table 3:** Adverse events summary

**n (%)**	**Pembrolizumab + Epacadostat** ***n* = 34**	**Pembrolizumab Monotherapy** ***n* = 19**	**EXTREME** ***n* = 34**
Any-grade all-cause AE	34 (100)	17 (89)	34 (100)
Grade 3–4	16 (47)	13 (68)	29 (85)
Led to discontinuation	3 (98)	2 (11)	7 (21)
Serious	12 (35)	8 (42)	12 (35)
Serious, led to discontinuation	2 (6)	1 (5)	1 (3)
Treatment-related AE^a^	28 (82)	12 (63)	34 (100)
Grade 3–4	8 (24)	3 (16)	28 (82)
Led to discontinuation	2 (6)	2 (11)	6 (18)
Serious	4 (12)	2 (11)	3 (9)
Serious, led to discontinuation	1 (3)	1 (5)	0
Died	0	0	0

*AE* Adverse event

^a^Determined by the investigator to be related to study treatment

**Table 4:** All-cause adverse events (> 10% in any group)

**n (%)**	**Pembrolizumab + Epacadostat** ***n* = 34**	**Pembrolizumab Monotherapy** ***n* = 19**	**EXTREME** ***n* = 34**
Fatigue	11 (32)	3 (16)	7 (21)
Rash	9 (26)	4 (21)	13 (38)
Diarrhea	7 (21)	2 (11)	7 (21)
Pruritus	7 (21)	0	6 (18)
Vomiting	7 (21)	0	5 (15)
Asthenia	6 (18)	2 (11)	8 (24)
Constipation	6 (18)	1 (5)	12 (35)
Dyspnea	6 (18)	1 (5)	2 (6)
Hypothyroidism	6 (18)	4 (21)	1 (3)
Lipase increased	6 (18)	0	4 (12)
Anemia	5 (15)	4 (21)	19 (56)
Insomnia	5 (15)	0	0
Nausea	5 (15)	2 (11)	17 (50)
Amylase increased	4 (12)	1 (5)	2 (6)
Back pain	4 (12)	0	2 (6)
Hemoptysis	4 (12)	0	1 (3)
AST increased	3 (9)	1 (5)	4 (12)
Cough	3 (9)	3 (16)	1 (3)
Decreased appetite	3 (9)	2 (11)	9 (26)
Headache	3 (9)	2 (11)	1 (3)
Pyrexia	3 (9)	0	5 (15)
Stomatitis	3 (9)	0	8 (24)
Weight decreased	3 (9)	2 (11)	9 (26)
ALT increased	2 (6)	1 (5)	5 (15)
Chest pain	2 (6)	2 (11)	2 (6)
Hypomagnesemia	2 (6)	0	9 (27)
Hypophosphatemia	2 (6)	2 (11)	2 (6)
Pain in extremity	2 (6)	2 (11)	1 (3)
Arthralgia	1 (3)	2 (11)	1 (3)
Hypertension	1 (3)	2 (11)	3 (9)
Hypokalemia	1 (3)	0	4 (12)
Neutrophil count decreased	1 (3)	0	8 (24)
Blood ALP increased	0	2 (11)	1 (3)
Dermatitis acneiform	0	0	14 (41)
Dysgeusia	0	0	4 (12)
Hyperglycemia	0	2 (11)	1 (3)
Mucosal inflammation	0	0	10 (29)
Nasopharyngitis	0	3 (16)	3 (9)
Neutropenia	0	0	13 (38)
Platelet count decreased	0	0	12 (35)
Skin lesion	0	2 (11)	0
Thrombocytopenia	0	0	9 (26)
WBC count decreased	0	0	7 (21)

*ALP* Alkaline phosphatase, *ALT* Alanine aminotransferase, *AST* Aspartate aminotransferase, *WBC* White blood cells

Treatment-related AEs were reported for 28 patients (82%) in the pembrolizumab plus epacadostat group, 12 (63%) in the pembrolizumab monotherapy group, and 34 (100%) in the EXTREME group (Table 3). Study treatment was discontinued due to treatment-related AEs in 2 patients (6%) in the pembrolizumab plus epacadostat group, 2 (11%) in the pembrolizumab monotherapy group, and 6 (18%) in the EXTREME group (Table 3). Grade 3–4 treatment-related AEs occurred in 8 patients (24%) in the pembrolizumab plus epacadostat group, 3 (16%) in the pembrolizumab monotherapy group, and 28 (82%) in the EXTREME group. The most common grade 3–4 treatment-related AEs in each group were lipase increased (*n* = 3 [9%]) in the pembrolizumab plus epacadostat group; arthralgia, diarrhea, fatigue, abnormal hepatic function, extremity pain, and peripheral edema (*n* = 1 [5%] for each) in the pembrolizumab monotherapy group; and anemia (*n* = 8 [24%]), neutropenia (*n* = 6 [18%]), platelet count decreased (*n* = 6 [18%]), neutrophil count decreased (*n* = 5 [15%]), and mucosal inflammation (*n* = 4 [12%]) with EXTREME (Table 5). Serious treatment-related AEs occurred in 4 patients (12%), 2 patients (11%), and 3 patients (9%), in the pembrolizumab plus epacadostat group, pembrolizumab monotherapy group, and the EXTREME group, respectively. Serious treatment-related AEs that occurred in > 1 patient in any treatment group were febrile neutropenia (EXTREME group, *n* = 2 [6%]) and pneumonitis (pembrolizumab plus epacadostat, *n* = 2 [6%]).

**Table 5:** Treatment-related adverse events^a^ of any grade (> 10% in any group) and corresponding events of grades 3–4^b^

**n (%)**	**Pembrolizumab + Epacadostat** ***n* = 34**		**Pembrolizumab Monotherapy** ***n* = 19**		**EXTREME** ***n* = 34**	
	**Any grade**	**Grade** **3–4**	**Any grade**	**Grade** **3–4**	**Any grade**	**Grade 3–4**
Fatigue	10 (29)	0	1 (5)	1 (5)	7 (21)	1 (3)
Rash	7 (21)	1 (3)	2 (11)	0	10 (29)	1 (3)
Hypothyroidism	6 (18)	0	3 (16)	0	0	0
Lipase increased	5 (15)	3 (9)	0	0	2 (6)	2 (6)
Pruritus	4 (12)	1 (3)	0	0	5 (15)	0
Anemia	3 (9)	1 (3)	1 (5)	0	18 (53)	8 (24)
Asthenia	2 (6)	1 (3)	1 (5)	0	7 (21)	2 (6)
Decreased appetite	2 (6)	0	0	0	7 (21)	0
Nausea	2 (6)	0	2 (11)	0	17 (50)	0
Vomiting	2 (6)	0	0	0	5 (15)	0
Blood creatinine increased	1 (3)	0	0	0	4 (12)	0
Hypomagnesaemia	1 (3)	0	0	0	8 (24)	2 (6)
Neutrophil count decreased	1 (3)	1 (3)	0	0	8 (24)	5 (15)
Stomatitis	1 (3)	0	0	0	8 (24)	0
Weight decreased	1 (3)	0	0	0	5 (15)	0
Dermatitis acneiform	0	0	0	0	14 (41)	1 (3)
Dysgeusia	0	0	0	0	4 (12)	0
Mucosal inflammation	0	0	0	0	9 (26)	4 (12)
Neutropenia	0	0	0	0	13 (38)	6 (18)
Paronychia	0	0	0	0	9 (26)	0
Platelet count decreased	0	0	0	0	11 (32)	6 (18)
Thrombocytopenia	0	0	0	0	7 (21)	3 (9)
WBC count decreased	0	0	0	0	7 (21)	1 (3)

*WBC* White blood cells

^a^Determined by the investigator to be related to study treatment

^b^No grade 5 treatment-related adverse events occurred within the study

Immune-mediated AEs were experienced by 8 patients (24%) in the pembrolizumab plus epacadostat group, 4 (21%) in the pembrolizumab monotherapy group, and 3 (9%) in the EXTREME group. The most frequently reported immune-mediated AEs in each group (Table 6) were hypothyroidism (*n* = 6 [18%]) and pneumonitis (*n* = 3 [9%]) in the pembrolizumab plus epacadostat group; hypothyroidism (*n* = 4 [21%]) and hyperthyroidism (*n* = 1 [5%]) with pembrolizumab monotherapy; and severe skin reactions (*n* = 2 [6%]) and hypothyroidism (*n* = 1 [3%]) with EXTREME. Grade 3–4 immune-mediated AEs occurred in 3 patients (9%) in the pembrolizumab plus epacadostat group, in 0 patients in the pembrolizumab monotherapy group, and in 2 patients (6%) in the EXTREME group. Grade 3–4 immune-mediated AEs in the pembrolizumab plus epacadostat group included grade 3 colitis (*n* = 1 [3%]), grade 3 nephritis (*n* = 1 [3%]), grade 3 severe skin reactions (*n* = 1 [3%]), grade 4 hepatitis (*n* = 1 [3%]), and grade 4 pneumonitis (*n* = 1 [3%]). For the EXTREME group 2 patients (6%) had grade 3 severe skin reactions.

**Table 6:** Immune-mediated adverse events^a^

**n (%)**	**Pembrolizumab + Epacadostat** ***n* = 34**	**Pembrolizumab Monotherapy** ***n* = 19**	**EXTREME** ***n* = 34**
Hypothyroidism	6 (18)	4 (21)	1 (3)
Pneumonitis	3 (9)	0	0
Colitis	1 (3)	0	0
Hepatitis	1 (3)	0	0
Hyperthyroidism	1 (3)	1 (5)	0
Nephritis	1 (3)	0	0
Severe skin reactions	1 (3)	0	2 (6)
Thyroiditis	1 (3)	0	0

^a^Immune-mediated adverse events were based on a list of terms specified by the sponsor and were included regardless of treatment attribution by investigators

**Fig. 2:**
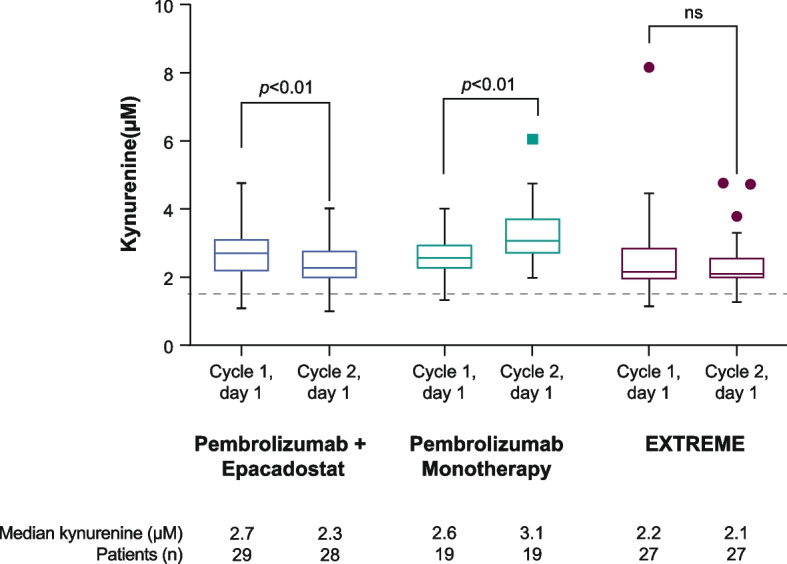
Circulating kynurenine levels^a,b^ in patients receiving pembrolizumab + epacadostat, pembrolizumab monotherapy, or EXTREME. Kynurenine levels at cycle 1, day 1, and cycle 2, day 1, were compared using a paired Student’s *t* test within each treatment arm. *ns* not significant. ^a^Blood samples were drawn before administration of any study drug on day 1 of cycle 1 and day 1 of cycle 2. ^b^Dotted line at 1.5 µM represents the median circulating kynurenine level in healthy subjects [12]

Median circulating kynurenine level was 2.7 µM at cycle 1 (*n* = 29) vs 2.3 µM at cycle 2 (*n* = 28) in the pembrolizumab plus epacadostat group (*P* < 0.01), 2.6 µM at cycle 1 (*n* = 19) vs 3.1 µM at cycle 2 (*n* = 19) in the pembrolizumab monotherapy group (*P* < 0.01), and 2.2 µM at cycle 1 (*n* = 27) vs 2.1 µM at cycle 2 (*n* = 27) in the EXTREME group (*P* = 0.36; no significant difference) (Fig. 2). The median kynurenine levels in all treatment groups were above the median level for healthy subjects (1.5 µM) [12].

## Discussion

In KEYNOTE-669, ORR of first-line pembrolizumab plus epacadostat (31%; 95% CI, 17%–49%) was similar to that of EXTREME (34%; 95% CI, 19%–52%) and pembrolizumab monotherapy (21%; 95% CI, 6%–46%). Note that due to the small sample size, the 95% CIs for ORR are wide for each treatment group and substantially overlapped. Three patients (9%) achieved a CR in the pembrolizumab plus epacadostat group versus zero patients in the pembrolizumab monotherapy and 2 patients (6%) in the EXTREME groups. In this study, the response rate for the chemotherapy-free regimen of pembrolizumab plus epacadostat (31% [11/35]; 3 CRs; 8 PRs) was comparable to responses observed in patients who received pembrolizumab plus epacadostat in the second-line or beyond setting for advanced solid tumors in the KEYNOTE-037/ECHO-202 study (40% [25/62]; 8 CRs; 17 PRs) [14], and more importantly, was similar to the response rate reported in KEYNOTE-048 for first-line pembrolizumab plus chemotherapy vs EXTREME (ORR 36% in both arms) [7]. The response rate of 21% with first-line pembrolizumab monotherapy in the current study was similar to that observed with pembrolizumab monotherapy in patients with R/M HNSCC in the phase 3 trials KEYNOTE-040 (15%) and KEYNOTE-048 (17%) [5, 7], supporting the validity of the response rate in this population to pembrolizumab monotherapy.

In this study, the ORR was higher with higher levels of PD-L1 expression in the pembrolizumab plus epacadostat (PD-L1 CPS ≥ 1, 34%; PD-L1 CPS ≥ 20, 40%) and pembrolizumab monotherapy groups (PD-L1 CPS ≥ 1, 18%; PD-L1 CPS ≥ 20, 33%). Similar results were seen in both KEYNOTE-040 and KEYNOTE-048, where higher levels of PD-L1 expression were associated with a higher proportion of patients having an objective response with pembrolizumab treatment [5, 7]. In KEYNOTE-040, the response rate was 17% in the PD-L1 CPS ≥ 1 population [5]. In KEYNOTE-048, response rates were 19% and 23% in the PD-L1 CPS ≥ 1 and CPS ≥ 20 populations, respectively [7].

The combination of pembrolizumab plus epacadostat in the current study was tolerable, with a safety profile comparable to pembrolizumab monotherapy and no new safety concerns identified. The incidence of grade 3/4 treatment-related AEs was lower with both pembrolizumab regimens, pembrolizumab plus epacadostat (24%) and pembrolizumab monotherapy (16%) vs EXTREME (82%) in patients with previously untreated R/M HNSCC. The incidence of grade ≥ 3 treatment-related AEs with pembrolizumab plus epacadostat in the present study (24%) was similar to that reported in KEYNOTE-037/ECHO-202 (24%) [14]. Fatigue was the most common treatment-related AE in patients receiving pembrolizumab plus epacadostat in this study (29%), which was consistent with a phase 3 trial of epacadostat plus pembrolizumab in patients with unresectable or metastatic melanoma [15]. The most common treatment-related AEs reported with pembrolizumab in KEYNOTE-040 and KEYNOTE-048 were hypothyroidism (13% in both) and fatigue (13% and 14%) [5, 7]. Hypothyroidism was also identified as the most common treatment-related AE with pembrolizumab monotherapy in this study, reported by 16% of patients.

The 100-mg twice-daily dose of epacadostat in combination with pembrolizumab was established as the recommended dose for further study based on the results of the phase 1/2 KEYNOTE-037/ECHO-202 study [14]. Additionally, pharmacodynamic analysis in this study showed that epacadostat appeared to limit the increase in kynurenine levels associated with pembrolizumab treatment but did not reduce levels to that of healthy volunteers [12]. The decrease in circulating kynurenine levels observed in patients receiving epacadostat is consistent with the mechanism of action of epacadostat as an IDO1 inhibitor, and with the findings of a phase 1 study of epacadostat monotherapy which has shown decreased kynurenine levels in patients with solid tumors [12] However, the results of a retrospective pooled analysis of epacadostat clinical studies has suggested that epacadostat at a dose of 100 mg twice daily when used in combination with anti–PD-1 inhibitors is not sufficient to normalize kynurenine levels, a finding also supported by the current analysis [16]. The authors hypothesized that anti–PD-1–induced upregulation of interferon gamma production may in turn upregulate IDO1, and consequently optimal inhibition of IDO1 in the presence of a PD-1 inhibitor may require doses of epacadostat ≥ 600 mg twice daily. The increase in circulating kynurenine levels observed with pembrolizumab in the current study is consistent with other analyses that have reported an increase in kynurenine levels in patients with solid tumors receiving PD-1 inhibitors [17, 18]. High levels of kynurenine have also been associated with poor prognosis in solid tumors [18]. Investigating higher doses of epacadostat in combination with pembrolizumab may therefore be warranted to investigate if further reductions in kynurenine levels are associated with improved outcome.

Limitations of the present study include the small sample size and short duration of follow-up owing to early discontinuation of enrollment. No biomarker analyses based on HPV status, PD-L1 expression, or other subgroups were performed because of the small subgroup sizes. Additionally, because of the early enrollment discontinuation, the study was not powered to show differences in ORR. Also, based on ORR evaluations at 9 weeks, it is uncertain that the observed differences are meaningful or will translate into clinical benefit based on time-to-event endpoints such as OS. Further, the incomplete target inhibition, as measured by plasma kynurenine, suggest additional testing of higher doses of epacadostat is needed when combined with checkpoint inhibitors. The dynamics of combination immunotherapy responses were not further evaluated in this trial, and time-to-event endpoints were not mature or considered in the decision to terminate the study.

Following the outcome of KEYNOTE-252, which did not meet the prespecified endpoint, several studies investigating combination therapy with an IDO1 inhibitor and an immune checkpoint inhibitor have been halted [19]. However, there are some ongoing phase 3 studies, such as nivolumab in combination with linrodostat (BMS-986205), a selective IDO-1 inhibitor, for the treatment of muscle-invasive bladder cancer (NCT03661320) and previously untreated metastatic/unresectable melanoma (NCT03329846).

## Conclusion

The results of the prematurely discontinued KEYNOTE-669/ECHO-304 study showed that pembrolizumab plus epacadostat provided a similar ORR compared with EXTREME and pembrolizumab monotherapy and demonstrated a manageable safety profile in patients with R/M HNSCC.

### Supplementary Information

**Additional file 1: **Summary of Protocol Amendments.** Supplementary Table 1.** Institutional Review Board or Ethics Committee of Each Participating Site. **Supplementary Table 2.** Summary of key changes to the protocol.

## Data Availability

Merck Sharp & Dohme LLC, a subsidiary of Merck & Co., Inc., Rahway, NJ, USA (MSD) is committed to providing qualified scientific researchers access to anonymized data and clinical study reports from the company’s clinical trials for the purpose of conducting legitimate scientific research. MSD is also obligated to protect the rights and privacy of trial participants and, as such, has a procedure in place for evaluating and fulfilling requests for sharing company clinical trial data with qualified external scientific researchers. The MSD data sharing website (available at: http://engagezone.msd.com/ds_documentation.php) outlines the process and requirements for submitting a data request. Applications will be promptly assessed for completeness and policy compliance. Feasible requests will be reviewed by a committee of MSD subject matter experts to assess the scientific validity of the request and the qualifications of the requestors. In line with data privacy legislation, submitters of approved requests must enter into a standard data-sharing agreement with MSD before data access is granted. Data will be made available for request after product approval in the US and EU or after product development is discontinued. There are circumstances that may prevent MSD from sharing requested data, including country or region-specific regulations. If the request is declined, it will be communicated to the investigator. Access to genetic or exploratory biomarker data requires a detailed, hypothesis-driven statistical analysis plan that is collaboratively developed by the requestor and MSD subject matter experts; after approval of the statistical analysis plan and execution of a data-sharing agreement, MSD will either perform the proposed analyses and share the results with the requestor or will construct biomarker covariates and add them to a file with clinical data that is uploaded to an analysis portal so that the requestor can perform the proposed analyses.

## Abbreviations

5-FU: 5-Fluorouracil
AE: Adverse event
AUC: Area under the curve
CI: Confidence interval
CPS: Combined positive score
CR: Complete response
ECOG: Eastern Cooperative Oncology Group
HNSCC: Head and neck squamous cell carcinoma
HPV: Human papillomavirus
IDO1: Indoleamine 2,3 dioxygenase 1
IV: Intravenous
ORR: Objective response rate
OS: Overall survival
PD-1: Programmed death-1
PD-L1: Programmed death ligand 1
PR: Partial response
Q3W: Every 3 weeks
RECIST v1.1: Response Evaluation Criteria in Solid Tumors version 1.1
R/M: Recurrent/metastatic
