# Supplementary material for: Pembrolizumab plus epacadostat in patients with recurrent/metastatic head and neck squamous cell carcinoma (KEYNOTE-669/ECHO-304): a phase 3, randomized, open-label study
Source: BMC Cancer. 2024 Jul 25;23(Suppl 1):1254. doi: 10.1186/s12885-023-11316-0 (PMC11270762; doi:10.1186/s12885-023-11316-0)
Supplement: Supplementary file 1 — Additional file 1: Summary of Protocol Amendments. Supplementary Table 1. Institutional Review Board or Ethics Committee of Each Participating Site. Supplementary Table 2. Summary of key changes to the protocol. [file 12885_2023_11316_MOESM1_ESM.docx]

**SUPPLEMENTARY MATERIALS**

**Summary of Protocol Amendments**

*Rationale for Amendments*

The external data monitoring committee for the KEYNOTE-252 study determined that the study did not meet the prespecified endpoint of improvement in progression-free survival for the combination of pembrolizumab and epacadostat compared to pembrolizumab and placebo in patients with unresectable or metastatic melanoma. The external data monitoring committee further determined that the overall survival endpoint was not expected to reach statistical significance. Of note, no new safety concerns were identified with the pembrolizumab plus epacadostat combination compared with pembrolizumab monotherapy. Enrollment in the KEYNOTE-669 study was permanently stopped on May 2, 2018, as a strategic decision. For participants who were considered to be obtaining ongoing clinical benefit, study treatment could be continued at the discretion of the investigator after a discussion with the participant of the observed results from the KEYNOTE-252 study. The protocol provides a complete list of changes. Some of the key changes to the KEYNOTE-669 study protocol are included in Supplementary Table 2.

**Supplementary Table 1.** Institutional Review Board or Ethics Committee of Each Participating Site

| **Country** | **Site** | **Principal investigator** | **Site name** | **IRB/EC name** |
| --- | --- | --- | --- | --- |
| USA | 0152 | Firas Badin | Baptist Healthcare System. Central Baptist Hospital | Baptist Health Institutional Review Board |
| USA | 0155 | Barbara Ann Burtness | Yale Cancer Center | Western Institutional Review Board |
| USA | 0156 | Steven C. Buck | Oklahoma Cancer Specialists and Research Institute | Western Institutional Review Board |
| USA | 0159 | Patrick Wayne Cobb | St. Vincent Healthcare Frontier Cancer Center | Western Institutional Review Board |
| USA | 0174 | Antonio Jimeno | University of Colorado Cancer Center | Western Institutional Review Board |
| USA | 0176 | Rom Leidner | Providence Health & Services, Providence Portland Medical Center | Providence Health and Services Institutional Review Board |
| USA | 0177 | Prakash Neupane | University of Kansas Cancer Center | Western Institutional Review Board |
| USA | 0182 | John Weis | Huntsman Cancer Institute | University of Utah Institutional Review Board |
| USA | 0183 | Thomas S Stanton | St. Joseph Heritage Healthcare | Western Institutional Review Board |
| USA | 0184 | Laura Stampleman | Pacific Cancer Care | Copernicus Group Independent Review Board |
| USA | 0191 | Jonathan Riess | UC Davis Comprehensive Cancer Center | UC Davis-Institutional Review Board Administration |
| USA | 0192 | Meaghan O'Malley | Virginia Mason Medical Center | Western Institutional Review Board |
| USA | 0193 | Emrullah Yilmaz | The University of New Mexico Comprehensive Cancer Center | Western Institutional Review Board |
| USA | 0194 | Steven McCune | Northwest Georgia Oncology Centers PC | Western Institutional Review Board |
| Canada | 0200 | Felix Couture | CHUQ - Pavillion l'Hotel Dieu de Quebec | Jewish General Hospital Research Ethics Committee |
| Canada | 0203 | Lillian L Siu | Princess Margaret Cancer Centre | OCREB - Ontario Cancer Research Ethics Board |
| Canada | 0208 | Sebastien J Hotte | Juravinski Cancer Centre | OCREB - Ontario Cancer Research Ethics Board |
| Canada | 0210 | Wilson H Miller | Jewish General Hospital | Jewish General Hospital Research Ethics Committee |
| Australia | 0250 | Marcin Dzienis | Gold Coast University Hospital | Royal Brisbane and Women’s Hospital Human Research Ethics Committee |
| Australia | 0252 | Baerin Houghton | MNCCI Port Macquarie Base Hospital | Royal Brisbane and Women’s Hospital Human Research Ethics Committee |
| Australia | 0253 | Brett Hughes | Royal Brisbane and Women’s Hospital | Royal Brisbane and Women’s Hospital Human Research Ethics Committee |
| Australia | 0255 | Michael Joseph Boyer | Chris OBrien Lifehouse | Royal Brisbane and Women’s Hospital Human Research Ethics Committee |
| Japan | 0300 | Makoto Tahara | National Cancer Center Hospital East | National Cancer Center Institutional Review Board |
| Japan | 0301 | Shunji Takahashi | The Cancer Institute Hospital of JFCR | Institutional Review Board of Cancer Institute Hospital of JFCR |
| Japan | 0302 | Nobuhiko Oridate | Yokohama City University Hospital | Yokohama City University Hospital Institutional Review Board |
| Japan | 0303 | Yasushi Shimizu | Hokkaido University Hospital | Hokkaido University Hospital Institutional Review Board |
| Japan | 0304 | Kaoru Tanaka | Kindai University Hospital | Kindai University Hospital Institutional Review Board |
| Japan | 0306 | Koji Matsumoto | Hyogo Cancer Center | Hyogo Cancer Center Institutional Review Board |
| Japan | 0307 | Tsutomu Ueda | Hiroshima University Hospital | Hiroshima University hospital Institutional Review Board |
| Japan | 0308 | Tomoya Yokota | Shizuoka Cancer Center Hospital and Research Institute | Shizuoka Cancer Center Institutional Review Board |
| Japan | 0309 | Tomoko Yamazaki | Miyagi Cancer Center | Miyagi Cancer Center Institutional Review Board |
| Japan | 0310 | Tomokazu Yoshizaki | Kanazawa University Hospital | Kanazawa University Hospital Institutional Review Board |
| Japan | 0315 | Hiroki Hara | Saitama Cancer Center | Saitama Cancer Center Institutional Review Board |
| Japan | 0316 | Hiroo Imai | Tohoku University Hospital | Tohoku University Hospital Institutional Review Board |
| Japan | 0317 | Hironaga Satake | Kobe City Medical Center General Hospital | Kobe City Medical Center General Hospital Institutional Review Board |
| Japan | 0318 | Torahiko Nakashima | National Hospital Organization Kyushu Medical Center | National Hospital Organization Kyushu Medical Center Institutional Review Board |
| South Korea | 0400 | Sung-Bae Kim | Asan Medical Center | Asan Medical Center Institutional Review Board/IEC |
| South Korea | 0401 | Bhumsuk Keam | Seoul National University Hospital | Seoul National University Hospital Institutional Review Board |
| South Korea | 0402 | Byoung Chul Cho/Hye Ryun Kim | Severance Hospital Yonsei University Health System | Yonsei University Health System, Severance Hospital Institutional Review Board |
| Taiwan | 0450 | Chia-Jui Yen | National Cheng Kung University Hospital | National Cheng Kung University Hospital Ethics Review Committee |
| Taiwan | 0451 | Shau-Hsuan Li/Tai-Jan Chiu | Chang Gung Medical Foundation. Kaohsiung Branch | Chang Gung Medical Foundation |
| Taiwan | 0452 | Ruey-Long Hong | National Taiwan University Hospital | National Taiwan University Hospital - Research Ethics Committee |
| Austria | 0550 | Martin Burian | Ordensklinikum Linz | Ethikkommission der Medizinischen Fakultät der JKU |
| Austria | 0551 | Thorsten Fuereder | Allgemeines Krankenhaus der Stadt Wien | Ethikkommission der Medizinischen Fakultät der JKU |
| Austria | 0552 | Richard Greil | Landeskrankenhaus Salzburg | Ethikkommission der Medizinischen Fakultät der JKU |
| Austria | 0553 | Dietmar Thurnher | Landeskrankenhaus - Universitatsklinikum Graz | Ethikkommission der Medizinischen Fakultät der JKU |
| Hungary | 0800 | Janos Revesz | Borsod-Abauj-Zemplen Megyei Korhaz es Egyetemi OktatoKorhaz | Egeszsegugyi Tudomanyos Tanacs |
| Hungary | 0803 | Tibor Csoszi | Jasz Nagykun Szolnok Megyei Hetenyi Geza Korhaz Rendelointezet | Egeszsegugyi Tudomanyos Tanacs |
| Poland | 0902 | Malgorzata Chudzik | Mazowiecki Szpital Onkologiczny | KB przy OIL w Szczeczinie. Komisja Bioetyczna przy ORL Wielkopolskiej Izby Lekarskiej |
| Poland | 0904 | Boguslawa Karaszewska | Przychodnia Lekarska Komed | KB przy OIL w Szczeczinie.  Komisja Bioetyczna przy ORL Wielkopolskiej Izby Lekarskiej |
| Poland | 0913 | Malgorzata Talerczyk | Zachodniopomorskie Centrum Onkologii | KB przy OIL w Szczeczinie. Komisja Bioetyczna przy ORL Wielkopolskiej Izby Lekarskiej |
| Spain | 0950 | Irene Brana | Hospital General Universitari Vall d’Hebron | Hospital General Universitario Ramon y Cajal Ctra. De Colmenar Viejo km. 9.1 CEIC - Area 4 Madrid, Madrid 28034 Spain Hospital General Universitario Ramon y Cajal Ctra. De Colmenar Viejo km. 9.1 CEIC - Area 4 Madrid, Madrid 28034 Spain CEIC Hospital Universitario Ramon y Cajal Carretera de Colmenar Viejo. Kilometro 9.1 Madrid, Madrid 28034 Spain CEIM Hospital General Universitario Gregorio Maranon Calle Dr. Esquerdo 46 Madrid, Madrid 28007 Spain |
| Spain | 0952 | Beatriz Cirauqui Cirauqui | Hospital Germans Trias i Pujol. ICO de Badalona | Hospital General Universitario Ramon y Cajal CEIC Hospital Universitario Ramon y Cajal Carretera de Colmenar Viejo, Madrid, Spain CEIM Hospital General Universitario Gregorio Maranon Madrid, Spain |
| Spain | 0956 | Ainara Soria | Hospital Ramon y Cajal | Hospital General Universitario Ramon y Cajal Madrid,  Spain Hospital General Universitario Ramon y Cajal Madrid, Spain CEIC Hospital Universitario Ramon y Cajal Madrid, Spain CEIM Hospital General Universitario Gregorio Maranon Madrid,  Spain |
| Spain | 0958 | Lara Iglesias Docampo | Hospital Universitario 12 de Octubre | Hospital General Universitario Ramon y Cajal Madrid, Spain Hospital General Universitario Ramon y Cajal Madrid, Spain CEIC Hospital Universitario Ramon y Cajal Carretera de Colmenar Viejo.  Madrid, Spain CEIM Hospital General Universitario Gregorio Maranon Madrid, Spain |
| Spain | 0959 | Ignacio Delgado | Hospital Infanta Cristina | Hospital General Universitario Ramon y Cajal Madrid,  Spain Hospital General Universitario Ramon y Cajal Madrid,  Spain CEIC Hospital Universitario Ramon y Cajal Madrid, Spain CEIM Hospital General Universitario Gregorio Maranon Madrid, Spain |
| Spain | 0960 | Julio Lambea | Hospital Clinico Lozano Blesa | Hospital General Universitario Ramon y Cajal Madrid,  Spain Hospital General Universitario Ramon y Cajal Madrid, Spain CEIC Hospital Universitario Ramon y Cajal Carretera de Colmenar Madrid,  Spain CEIM Hospital General Universitario Gregorio Maranon |
| Spain | 0961 | Manuel Chaves Conde | Hospital de Nuestra Senora de Valme | Hospital General Universitario Ramon y Cajal.  CEIC Hospital Universitario Ramon y Cajal. CEIM Hospital General Universitario Gregorio Maranon. |
| Turkey | 1000 | Sercan Aksoy | Hacettepe Universitesi Tip Fakultesi Hastanesi Hacettepe Üniversitesi ç Hastaliklari ABD Kanser Enstitüsü, Medikal Onkoloji Bilim Dal, Sihhiye Ankara, | Inonu Universitesi Malatya Klinik |
| Turkey | 1001 | Meltem Ekenel | Istanbul Universitesi Onkoloji Enstitusu | Inonu Universitesi Malatya Klinik |
| Turkey | 1002 | Irfan Cicin | Trakya Uni. Tip Fakultesi Trakya UTF Onkoloji Bilim Dali Balkan Yerleskesi | Inonu Universitesi Malatya Klinik |
| Turkey | 1003 | Hakan Harputluoglu | Inonu Universitesi Turgut Ozal Tip Merkezi | Inonu Universitesi Malatya Klinik |
| Turkey | 1016 | Timucin Cil | Adana Sehir Hastanesi D Blok Zemin kat Tibbi Onkoloji Polilklinigi | Inonu Universitesi Malatya Klinik |
| Turkey | 1017 | Ahmet Bilici | Medipol Universite Hastanesi | Inonu Universitesi Malatya Klinik |
| Turkey | 1018 | Ruchan F Uslu/Erhan Gokmen | Ruchan F Uslu/Erhan Gokmen Ege Universitesi Tip Fakultesi Hastanesi. Tulay Aktas Onkoloji Hastanesi Tibbi Onkoloji Bilim. | Inonu Universitesi Malatya Klinik |
| UK | 1050 | Anna Thompson | North Middlesex Hospital Sterling Way. | NRES Committee London Central and  London Central Research Ethics Committee |
| UK | 1051 | Martin David Forster | University College London Hospital NHS Foundation Trust | NRES Committee London Central and  London Central Research Ethics Committee |
| UK | 1054 | Petra Jankowska | Musgrove Park Hospital | NRES Committee London Central and  London Central Research Ethics Committee |
| UK | 1058 | Kevin Harrington | The Royal Marsden Foundation Trust Renal and Melanoma Unit | NRES Committee London Central and  London Central Research Ethics Committee |
| UK | 1059 | Kevin Harrington | Royal Marsden NHS Foundation Trust Head Neck and Thyroid Unit | NRES Committee London Central and  London Central Research Ethics Committee |
| Portugal | 1200 | Jose Dinis | Inst. Portugues de Oncologia de Porto Francisco Gentil EPE | CEIC - Comissao de Etica para a Investigacao Clinica |
| Portugal | 1201 | Leonor Castro Abreu Ribeiro | Leonor Castro Abreu Ribeiro Centro Hospitalar Lisboa Norte EPE - Hospital de Santa Maria Avenida Professor Egas Moniz Servico de Oncologia | CEIC - Comissao de Etica para a Investigacao Clinica |
| Portugal | 1202 | Margarida Ferreira | Inst. Portugues de Oncologia de Lisboa Francisco Gentil EPE | CEIC - Comissao de Etica para a Investigacao Clinica |
| Portugal | 1203 | Margarida Teixeira | Inst. Portugues de Oncologia de Coimbra Francisco Gentil EPE | CEIC - Comissao de Etica para a Investigacao Clinica |
| Italy | 1450 | Raffaele Cavina | Istituto Clinico Humanitas - Cancer Center | Comitato Etico Indipendente Istituto Clinico Humanitas |
| Italy | 1451 | Franco Nole | IEO Istituto Europeo di Oncologia | Comitato Etico degli IRCCS Istituto Europeo |

If the original investigator has been replaced, that investigator’s name is followed by a slash (/) and the replacement investigator’s name.

**Supplementary Table 2.** Summary of key changes to the protocol

| **Section** | **Description of Change** | **Brief Rationale** |
| --- | --- | --- |
| **Synopsis, Schedule of Activities, Second Course Phase, Overall Design, Tumor Imaging and Assessment of Disease, End of Treatment and Follow-up Tumor Imaging** | Modifications to clarify the following throughout the protocol:   - Enrollment was stopped as of May 2, 2018 - After the first imaging assessment for efficacy analysis, participants could choose to discontinue from the study or continue study treatment as per protocol after participant discussion with the investigator - All study efficacy procedures will stop after Week 9 and thereafter be performed as per local standard of care - Imaging assessment will no longer be made by blinded independent central review, and that all disease progression will be assessed by the investigator based on RECIST 1.1 - The end of treatment scan will be performed for participants who discontinue study treatment before the first scheduled on-study imaging for efficacy analysis - Note on recording of all post-study anticancer therapy was deleted | A strategic decision was made to permanently stop enrolment in KEYNOTE-669 following the findings of the external data monitoring committee for the KEYNOTE-252 study of pembrolizumab + epacadostat in melanoma. Participants enrolled in KEYNOTE-669 were given the option to discontinue from the study or continue study treatment. The study remained open to allow participants access to study treatment and to allow collection of preliminary efficacy data in this HNSCC indication. Efficacy procedures after Week 9 were no longer being mandated. |
| **4. Objectives/Hypotheses and Endpoints** | All text referring to progression-free survival, overall survival, and duration of response was deleted. Evaluation of objective response rate based on RECIST 1.1 as assessed by the investigator was included as the primary objective. All other efficacy endpoints will no longer be collected after Week 9. Safety endpoints remained unchanged. | The study scope was reduced to collect preliminary efficacy data. |
| **1. Synopsis** | The estimated duration of the trial was decreased from 50 to 41 months. | The study scope was reduced to collect preliminary efficacy data. |
| **1. Synopsis** | Text relating to follow-up of participants who discontinue treatment for reasons other than disease progression was replaced with text relating to discontinuation from the study after the Safety Follow-up Visit. | The final visit in the study will be the Safety Follow-Up Visit and there will be no follow-up for survival status. Participants currently in follow-up or in survival follow-up are considered to have completed the study. However, standard safety reporting will continue, as applicable. |
| **5.1.1. Data Monitoring Committee** | Text referring to an interim analysis was removed. | The study scope was reduced to collect preliminary efficacy data. |
| **5.4.1.1.2. Modified RECIST for Immune Based Therapeutics (irRECIST)** | Addition of text noting that this section is no longer applicable; iRECIST data will no longer be collected. Participants with radiographic disease progression as determined by RECIST 1.1 will discontinue from the study and be followed for safety monitoring. No confirmatory scans are required. | The study scope was reduced to collect preliminary efficacy data. |
| **Patient-Reported Outcomes** | Text removed regarding collection of patient- reported outcome data. | The study scope was reduced to collect preliminary efficacy data. |
